# Anti-Inflammatory Activity of Oligomeric Proanthocyanidins Via Inhibition of NF-κB and MAPK in LPS-Stimulated MAC-T Cells

**DOI:** 10.4014/jmb.2006.06030

**Published:** 2020-08-28

**Authors:** Xiao Ma, Ruihong Wang, Shitian Yu, Guicong Lu, Yongxiong Yu, Caode Jiang

**Affiliations:** Chongqing Engineering Research Centre for Herbivores Resource Protection and Utilization, College of Animal Science and Technology, Southwest University, Chonqing 400715, P.R. China

**Keywords:** Oligomeric proanthocyanidins, anti-inflammation, NF-κB, MAPK, MAC-T cell

## Abstract

Oligomeric proanthocyanidins (OPCs), classified as condensed tannins, have significant antioxidation, anti-inflammation and anti-cancer effects. This study was performed to investigate the anti-inflammatory effects of OPCs and the mechanism underlying these effects in lipopolysaccharide (LPS)-stimulated bovine mammary epithelial cells (MAC-T). Real-time PCR and ELISA assays indicated that OPC treatment at 1, 3 and 5 μg/ml significantly reduced the mRNA and protein, respectively, of oxidant indicators cyclooxygenase-2 (COX-2) (*p* < 0.05) and inducible nitric oxide synthase (iNOS) (*p* < 0.01) as well as inflammation cytokines interleukin (IL)-6 (*p* < 0.01), IL-1β (*p* < 0.01) and tumor necrosis factor-α (TNF-α) (*p* < 0.05) in LPS-induced MAC-T cells. Moreover, OPCs downregulated LPSinduced phosphorylation of p65 and inhibitor of nuclear factor kappa B (NF-κB) (IκB) in the NF-κB signaling pathway (*p* < 0.01), and they inhibited p65 translocation from the cytoplasm to the nucleus as revealed by immunofluorescence test and western blot. Additionally, OPCs decreased phosphorylation of p38, extracellular signal regulated kinase and c-jun NH_2_-terminal kinase in the MAPK signaling pathway (*p* < 0.01). In conclusion, the anti-inflammatory and antioxidant activities of OPCs involve NF-κB and MAPK signaling pathways, thus inhibiting expression of pro-inflammatory factors and oxidation indicators. These findings provide novel experimental evidence for the further practical application of OPCs in prevention and treatment of bovine mastitis.

## Introduction

Bovine mastitis, an infectious disease common in the dairy farming industry, is mainly caused by pathogenic microorganisms. Bovine mastitis not only lowers milk quality and production, but also significantly increases the cost of treatment and management at dairy farms [1], which has led to adverse economic effects in the dairy industry over the last decades.

The major pathogens of mastitis include *Escherichia coli* and *Staphylococcus aureus*, which induce severe clinical and subclinical mastitis, respectively [1]. Lipopolysaccharide (LPS), a major component of the outer membrane of *E. coli*, is recognized as a virulence factor for bovine mastitis [2, 3]. In the canonical signaling pathway of inflammation, membrane Toll-like receptor 4 (TLR4) recognizes LPS stimulus via dimerization and activates inhibitor of nuclear factor kappa B (NF-κB) (IκB) kinase (IKK1/2) [2, 4]. IKK1/2 promotes phosphorylation of NF-κB and NF-κB-bound IκBs, leading to IκB degradation in proteasome via ubiquitination. The degradation of IκB frees NF-κB, which subsequently migrates into the nucleus to activate its target genes. TLR4 also activates mitogen-activated protein kinase (MAPK) pathway, which cross-talks with NF-κB pathway to participate in inflammatory responses [5, 6]. Activated NF-κB and MAPK signaling pathways induce transcription of inflammation cytokines, *e.g.*, interleukin (IL)-6 and IL-1β and inflammation mediators, such as inducible nitric oxide synthase (iNOS) and anti-oxidant indicators cyclooxygenase-2 (COX-2) [7].

Antibiotics are widely used for treatment of mastitis; however, antibiotic therapy for established mammary infection offers very limited effect [8]. Moreover, the excessive application of antibiotics is increasingly arousing worldwide concern over bacterial resistance and consumer health [9, 10]. To this end, there is an urgent need to develop new alternative treatments for bovine mastitis. As plant active ingredients are non-toxic and have medicinal and health benefits, their application in inflammation treatment has received widespread attention [11].

Proanthocyanidins (PCs), also referred to as condensed tannins, are mainly present in fruits, nuts, flowers and seeds of plants [12]. PCs are formed by condensation of flavan-3-ol subunits (catechin and epicatechin) [12]. Evidence from human, animal and culture cell studies has revealed the significant anti-inflammation, anti-oxidation, anti-tumor, anti-allergy, and anti-toxicity activities of PCs [13, 14]. PCs reportedly have potent anti-oxidative activity that immensely exceeds that of β-carotene as well as Vitamins C and E, and they can alleviate oxidative stress by modulating signaling pathways associated with phosphoinositide 3-kinase/Akt, MAPK, NF-κB and nuclear factor erythroid 2-related factor 2 [15]. Particularly, PCs extracted from grape seed ameliorate collagen-induced arthritis by modifying the TLR4-MyD88-NF-κB signaling pathway [16]. In addition, PCs downregulate NF-κB and AP-1 in LPS-activated RAW264.7 cells and mediate the MAPK pathway by inhibiting p38, extracellular signal-regulated kinase (ERK) and c-Jun N-terminal kinase (JNK) [17]. However, little information is available about immunomodulatory function of PCs in bovine mastitis.

Oligomeric proanthocyanidins (OPCs) are important secondary metabolites in plants and reportedly have a broad spectrum of bioactivities against oxidative stress and inflammation over polymers [12]. In this study, we thus investigated the anti-inflammatory effect of OPCs by evaluating their ability to inhibit inflammatory mediators and cytokine production in LPS-induced MAC-T cells. Then, we determined the molecular mechanism underlying OPCs anti-inflammatory activity.

## Materials and Methods

### Reagents and Chemicals

OPCs (CAS#: 4852-22-6) extracted from grape seeds were provided by Shanghai Yuanye (China). LPS and dexamethasone (DEX) were provided by Macleans (China). MAC-T cells were purchased from BMCC (China). Nuclear protein extraction kits and 3-(4,5-dimethylthiazol-2-yl)-2,5-diphenyltetrazole bromide (MTT) were purchased from Solarbio (China). Fluorescence quantification kits were provided by Takara (China). Primary antibodies against phosphorylated p65 (p-p65), p-ERK1/2 p-IκBα, p65, IκBα and ERK1/2 were obtained from Bioss (China), and antibodies against JNK1/2, p38, p-JNK1/2, and p-p38 were from Wanleibio (China). Dulbecco’s modified Eagle’s medium (DMEM) and fetal bovine serum (FBS) were purchased from Gibco (China). NF-κB Activation Nuclear Transport Test Kit and ELISA Kit were supplied by Bevotime (China) and Sinobestbio (China), respectively. Tris-buffered saline + Tween 20 (TBST) was supplied by Solarbio.

### Cell Culture and Treatment

MAC-T cells were cultured in DMEM basic containing 10% FBS, 100 U/ml penicillin and 100 μg/ml streptomycin at 37°C and 5% CO2 in an incubator. The cells were grown to logarithmic phase, or 90-100% confluency, and then the cells were co-treated with LPS (1 μg/ml) and various concentrations of OPCs (1, 3, and 5 μg/ml) for 24 h. The LPS concentration was applied as in the previous report by Wang *et al*. [18], and the OPC concentrations were chosen from cell viability data. The cells cultured in the medium containing LPS (1 μg/ml) without OPCs (1 μg/ml) and LPS with DEX (20 μg/ml), an anti-inflammatory drug, were used as controls.

### Cell Viability Assay

Cells were cultured in a 96-well plate until 90-100% confluency, and they were then administrated with OPCs at different concentrations (0, 1, 3, 5, 7, 10, 15, 20, and 40 μg/ml) in five replicates. After 24 h of incubation, the medium was replaced with 10 μl of MTT solution and 90 μl of DMEM followed by incubation for another 4 h. Then, the supernatant was discarded, and the cells were incubated in 110 μl formazan solution for 10 min. The absorbance was measured at 490 nm in xMark (Bio-Rad,  USA).

### Real-Time PCR

Cells were cultured and treated as previously described in a 12-well plate. Total RNA was extracted from cells by RNAiso, and cDNA was obtained using a Reverse Transcription System (Takara). Real-time PCR was conducted with TB Green Premix Ex Taq II (Takara), and primers are shown in Table 1. Melting curve analysis (60-95°C) was used for assessing amplification specificity. The PCR condition and cycling parameters were described recently [19]. Each reaction was performed in triplicate. Relative expression of each gene was calculated according to the 2^-ΔΔCt^ method with β-actin gene as an endogenous control [20].

### ELISA Assay for Inflammatory Cytokines and Mediators

Cells were cultured and treated as previously described in a 6-well plate. Cell protein was extracted using Total Protein Extraction Kits and quantified using BCA Protein Quantification Kits (Vazyme, China). The contents of TNF-α, IL-1β, IL-6, COX-2, and iNOS were assessed using commercial ELISA kits (Sinobestbio, China) according to the manufacturer’s instructions. Each ELISA reaction was performed in triplicate. The absorbance values were measured at 450 nm in xMarkTM (Bio-Rad).

### Western Blot Analysis for NF-κB and MAPKs

Cells were cultured and treated as previously described in a 6-well plate. Cell protein was extracted using Total Protein Extraction Kits, and quantified using BCA Protein Quantification Kits (Vazyme, China). For each sample, 20 μg protein was separated in 12% SDS-PAGEs. The protein bands were transferred to a polyvinylidene fluoride (PVDF) membrane and then blocked in Tris-buffered saline containing 0.05% Tween 20 and 5% bovine serum albumin for 2 h. The PVDF membranes were incubated at 4°C overnight with specific primary antibodies (1:1000 dilution in TBST) against p65, p-p65, IκBα, p-IκBα, p38, p-p38, JNK, p-JNK, ERK1/2, and p-ERK1/2. On the next day, the PVDF membranes were washed with TBST 5 times for 8 min each, and incubated with Rabbit Anti-Goat IgG/HRP (Bioss, China) antibody in 1:1000 dilution in TBST for 1 h at room temperature. After washing with TBST as described above, the protein bands were visualized with ECL western blotting detection reagent (Bioground, China). The experiments were repeated three times. β-actin protein was used as an endogenous control as in the previous report in MAC-T cells [20]. Band intensity normalized to β-actin was evaluated using Image Lab software (version 5.2.1, Bio-Rad).

### Measuring p65 Nuclear Translocation by Immunofluorescence and ELISA 

For immunofluorescence, MAC-T cells were cultured in 96-well plates until 5,000 cells per well, and they were treated as previously described. The cells were incubated with the primary antibody against p65, followed by incubation with Rabbit Anti-Goat IgG/Cy3 antibody (Bioss). The nuclei were stained blue with 4’, 6-diamidino-2-phenylindole (DAPI) and observed with an inverted fluorescent microscope (Leica, Germany).

For an ELISA test of p65 nuclear translocation, cells were cultured and treated as previously described in a 6-well plate. Nuclear protein was extracted using Nuclear Protein Extraction Kits, and was quantified using BCA Protein Quantification Kits (Vazyme, China). The content of NF-κB p65 in the nucleus was assessed using commercial ELISA kits as previously described.

### Statistical Analysis

All results are represented as mean ± standard error of mean (SEM). A t-test was performed between control and LPS without DEX and OPC treatments. One-way analysis of variance, followed by the Tukey post hoc test, was used to compare the significance among LPS combined with DEX and OPC treatments. Statistical significance was set to *p* < 0.05 or *p* < 0.01.

## Results

### Effect of OPCs on Cell Viability

**Fig. 1:**
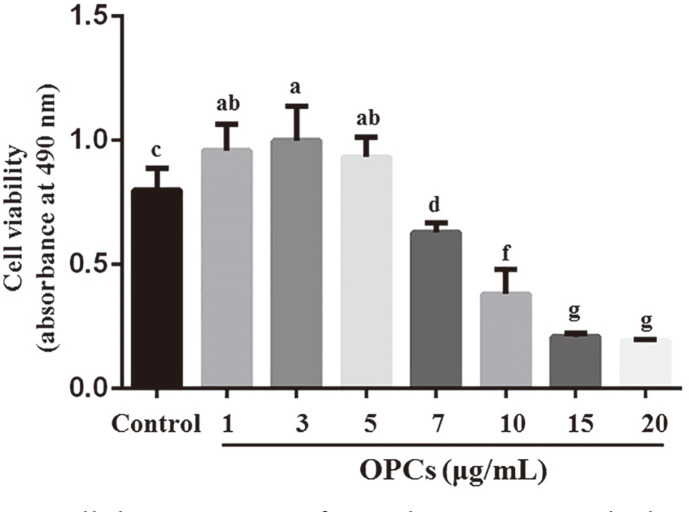
Cell viability of MAC-T cells by MTT assay after 24 h treatment with oligomeric proanthocyanidins (OPCs). Values represent means ± SEM of five independent experiments. Above bars, same letters indicated *p* > 0.05, different adjacent letters indicated *p* < 0.05, and different nonadjacent letters indicate *p* < 0.01.

OPC cytotoxicity was determined by MTT viability assays. MAC-T cells were treated with various concentrations of OPCs for 24 h. Compared to the control (0 μg/ml), OPC administration up to 7 μg/ml produced significant cytotoxicity to MAC-T cells in a dose-dependent manner (*p* < 0.05). However, viability of the cells treated with OPCs at concentrations of 1, 3 and 5 μg/ml was higher than that of the control (*p* < 0.05, Fig. 1). Therefore, the OPC concentrations of 1, 3, and 5 μg/ml were chosen for further research.

### Inhibition of OPCs on Expression of LPS-Stimulated Inflammatory Cytokines

First, the pro-inflammatory effect of LPS on MAC-T cells was tested based on mRNA expression of IL-6. As expected, significantly higher mRNA expression of IL-6 was detected in cells treated with LPS than in control cells (*p* < 0.01), but there was no difference in IL-6 production between LPS treatments at 1, 5, 10, and 20 μg/ml (*p* > 0.05, Fig. S1). Therefore, 1 μg/ml LPS was chosen for further investigation as previously reported [21].

**Fig. 2:**
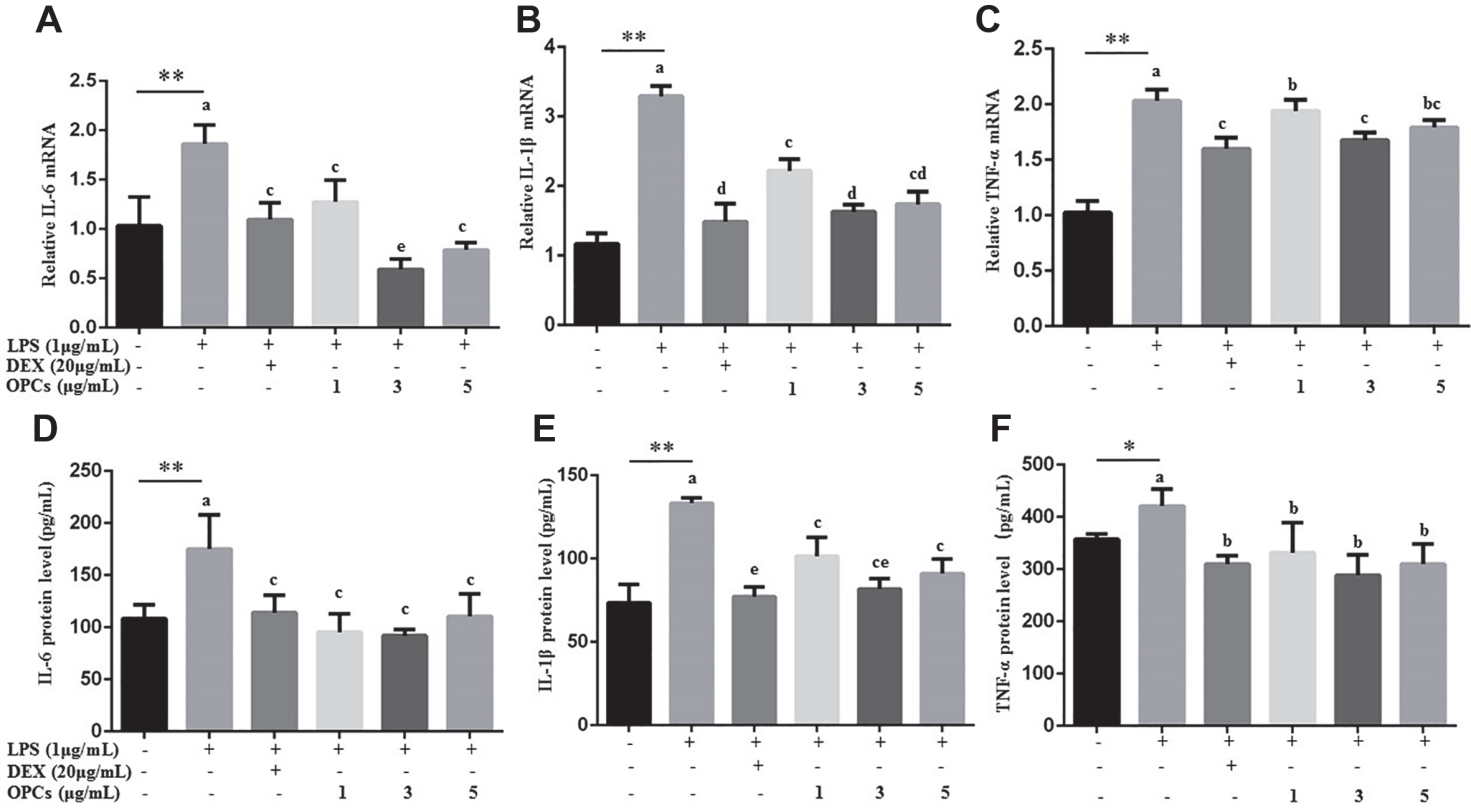
Expression examination of pro-inflammatory cytokines in MAC-T cells. Cells were treated with 1 μg/ml LPS for 24 h in combination with DEX (20 μg/ml) or OPCs (1, 3, and 5 μg/ml). Real-time PCR was used to detect mRNA levels (**A-C**) related to β-actin, and ELISA was used to detect protein levels (**D-F**) with normalized concentration total protein. Values represent means ± SEM of four independent experiments. Above bars, * and ** indicate significance at *p* < 0.05 and *p* < 0.01, respectively, between the control and the LPS treatment without DEX and OPCs. Among LPS in combination with DEX and OPCs treatments, same letters indicate *p* > 0.05, different adjacent letters indicate *p* < 0.05, and different nonadjacent letters indicate *p* < 0.01.

**Fig. 3:**
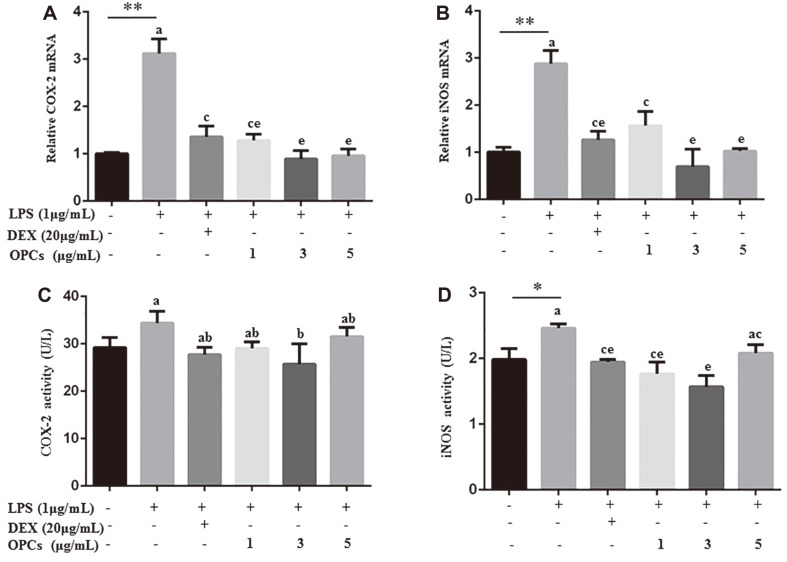
Expression analysis of COX2 and iNOS in MAC-T cells. Cells were incubated with 1 μg/ml LPS for 24 h in combination with DEX (20 μg/ml) or OPCs (1, 3, and 5 μg/ml). Real-time PCR was used to detect mRNA levels (**A and B**) with β-actin as an endogenous control, and ELISA was used to detect protein levels (**C and D**) with the protein concentration. Values represent means ± SEM of four independent. Above bars, * and ** indicate significance at *p* < 0.05 and *p* < 0.01, respectively, between the control and the LPS treatment without DEX and OPCs. Among LPS in combination with DEX and OPCs treatments, same letters indicate *p* > 0.05, different adjacent letters indicated *p* < 0.05, and different nonadjacent letters indicate *p* < 0.01.

Next, the anti-inflammatory effect of OPCs was tested by examining expression of inflammatory biomarkers with real-time PCR and ELISA. A significant rise in mRNA and protein levels of IL-6, IL-1β, and TNF-α was obtained in the LPS group compared to the untreated control group (*p* < 0.01, Fig. 2). After administration of OPCs, mRNA expression of IL-6 (*p* < 0.01), IL-1β (*p* < 0.01) and TNF-α (*p* < 0.05) was significantly attenuated with more prominent activity at 3 μg/ml dose, which showed better effect than DEX (Figs. 3A-3C). Furthermore, OPC administration inhibited protein expression of these inflammation biomarkers in LPS-stimulated MAC-T cells (Figs. 3D-3F), supporting the results of the qRT-PCR experiments.

**Fig. 4:**
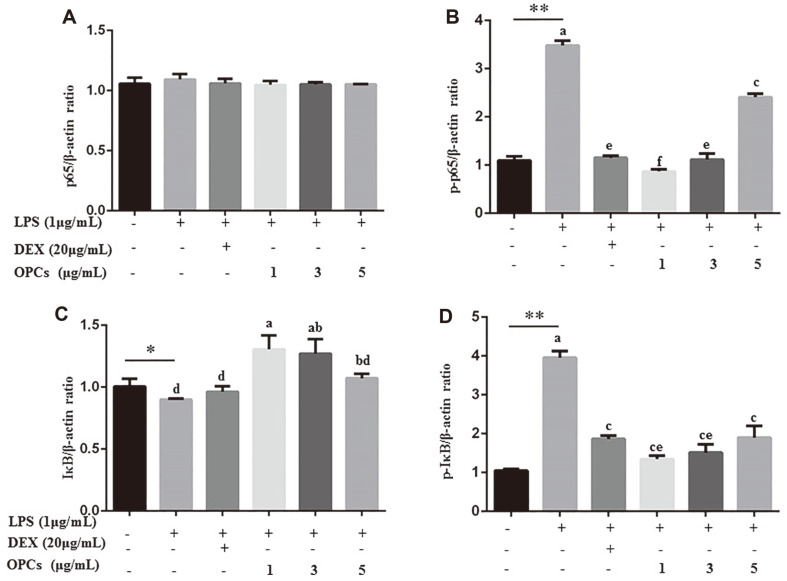
Detection of NF-κB activity in MAC-T cells by western blotting. Cells were incubated with 1 μg/ml LPS for 24 h in combination with DEX (20 μg/ml) or OPCs (1, 3, and 5μg/ml). Protein levels of p65 (**A**), p-p65 (**B**), IκB (**C**), and p-IκB (**D**) were relative to β-actin. Values represent means ± SEM of three independent experiments. Above bars, * and ** indicate significance at *p* < 0.05 and *p* < 0.01, respectively, between the control and the LPS treatment without DEX and OPCs. Among LPS in combination with DEX and OPCs treatments, same letters indicate *p* > 0.05, different adjacent letters indicate *p* < 0.05, and different nonadjacent letters indicate *p* < 0.01.

Additionally, the effect of OPCs on expression of oxidative stress indicators, *i.e.*, COX-2 and iNOS, was also examined. Both DEX and OPC treatments significantly downregulated LPS-induced mRNA abundance of COX-2 and iNOS in MAC-T cells (*p* < 0.01, Figs. 4A and 4B). ELISA showed that OPCs administration also inhibited the production of COX-2 and iNOS in LPS-stimulated MAC-T cells (Figs. 4C and 4D).

### Repression of OPCs on LPS-Stimulated NF-κB and MAPK Activation

**Fig. 5:**
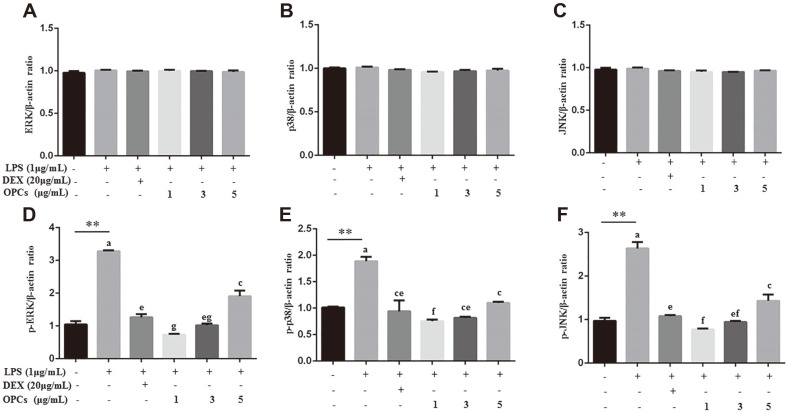
Detection of MAPKs activity in MAC-T cells by western blotting. Cells were incubated with 1 μg/ml LPS for 24 h in combination with DEX (20 μg/ml) or OPCs (1, 3, and 5 μg/ml). Protein levels of ERK1/2 (**A**), p38 (**B**), JNK (**C**), p-ERK1/2 (**D**), p-p38 (**E**), and p-JNK (**F**) were relative to β-actin. Values represent means ± SEM of three independent experiments. Above bars, ** indicate significance at *p* < 0.01 between the control and the LPS treatment without DEX and OPCs. Among LPS in combination with DEX and OPCs treatments, same letters indicate *p* > 0.05, different adjacent letters indicate *p* < 0.05, and different nonadjacent letters indicate *p* < 0.01.

We evaluated the influence of OPCs on NF-κB pathway, which was vital in the regulation of pro-infammatory cytokines. As shown in Figs. 5A andhere was no variation in protein levels of p65, a key member in the NF-κB pathway, between LPS, DEX, and OPCs treatments, but a significant decrease in phosphorylation of p65 was observed in cells treated with OPC (1, 3, and 5 μg/ml) and DEX (*p* < 0.01). Interestingly, LPS-inhibited proein levels of IκB, another key memeber in NF-κB pathway, were induced by OPC treatment, especially at concentrations of 1 and 3 μg/ml (*p* < 0.01, Fig. 4C). Consistent with our results, OPCs greatly inhibited LPS-increased phosphorylation of IκB in a dose-dependent manner (Fig. 4D). Also, OPC treatment significantly decreased the ratios of p-p65 and p-IκB to their total protein in a manner of dose-dependence (*p* < 0.01, Fig. S2).

We further examined expression of MAPK pathway, which is a critical cascade upstream NF-κB and pro-inflammatory mediators. Like the influence of OPCs on p-p65 and p-IκB, OPCs administration significantly attenuated LPS-induced activation of MAPK key members, including p-p38, p-JNK1/2, and p-ERK1/2 (*p* < 0.01), as revealed in both phosphorylation/β-actin (Fig. 5) and phosphorylation/total protein ratio (*p* < 0.01, Fig. S3).

### Prohibition of OPCs on LPS-Stimulated Nuclear Translocation of NF-κB p65

**Fig. 6:**
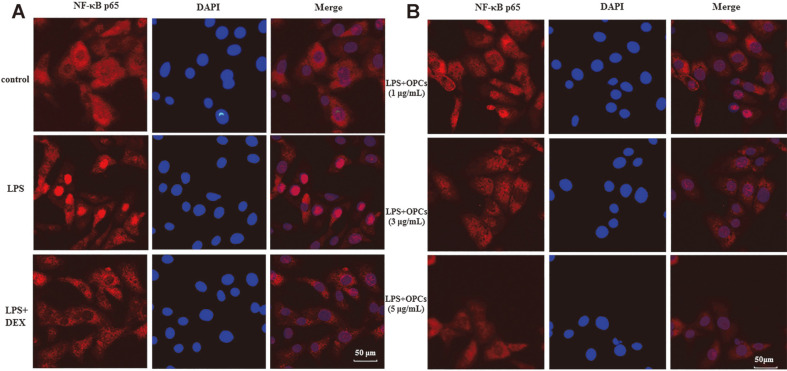
Immunofluorescence analysis of nuclear translocation of p65 in MAC-T cells. Cells stimulated with LPS (1 μg/ml) were cotreated with DEX (20 μg/ml) or OPCs (1, 3, and 5 μg/ml) for 24 h. p-p65 was labeled with fluorescence Cy3 (red), while nucleus was marked with fluorescence DAPI (blue).

We investigated nuclear translocation of p65 in LPS-challenged MAC-T cells under a fluorescence inverted microscope. Unlike the control group where red-fluorescent-labeled p65 was found in the cytoplasm, LPS treatment led to the accumulation of p65 in the blue-labeled nuclei (Fig. 6A). However, LPS-enhanced translocation of p65 was prohibited by OPCs treatment at concentrations of 1, 3, and 5 μg/ml (Fig. 6B), similar to the effect of DEX shown in Fig. 6A.

The fraction of NF-kB p65 in the nucleus was examined. As shown in Fig. S4, LPS stimulation led to higher levels of NF-κB p65 in the nucleus over the non-treatment control (*p* < 0.01). However, administration with 1, 3, and 5 mg/ml of OPCs markedly reduced LPS-induced nuclear content of p65 (*p* < 0.01), supporting the results of immunofluorescence experiments.

## Discussion

Dairy mastitis is one of the main reasons for cow disposal [22]. The bovine mammary epithelium serves as a crucial site for lactation and the first line of defense in the bacterial invasion process, thus mammary epithelial cells are considered to be important for investigating inflammatory responses. To this end, the MAC-T cell line was used in this study for elucidation of the effects of OPCs on bovine mammary inflammation because of their similar biological responses as primary cells of the bovine mammary epithelium [23]. It has been reported that LPS, the main component of cell membranes in *E. coli*, triggers a comprehensive immune response involving cytokine and chemokine expression induced in MAC-T cells via TLR4/NF-κB and MAPK cascades [24, 25]. Our results showed that LPS at 1 μg/ml triggered inflammatory repsonse in MAC-T cells (Fig. S1), consistent with Wang *et al*. [18]. Thus, 1 μg/ml LPS and 1, 3, and 5 μg/ml OPCs without cytotoxicity to MAC-T cells were determined for in vivo experiments (Fig. 1).

Previous work demonstrated an important role of cytokines in promoting cellular inflammation in response to bacterial pathogens [26]. LPS-induced pro-inflammatory IL-6, TNF-α and IL-1β result in serious damage to bovine udder gland tissues, and PCs attenuate these cytokines in human periodontal ligament fibroblasts, mouse autoimmune arthritis and RAW264.7 macrophages [16, 17, 27]. The procyanidins’ metabolites , i.e., catechin and epicatechin, can also alleviate inflammatory response. Epicatechin has been shown to inhibit inflammation factors including TNF-α and IL-6 in mouse colitis and rat renal inflammation [28, 29], while catechin can restrain the LPS-induced inflammation in BV-2 cells and human dental pulp cells [30, 31]. Consistent with these findings, our results also indicated that epicatechin alleviated inflammatory response on MAC-T cell inflammation (in preparation for publication). All in all, our data showed that OPC administration attenuated mRNA and protein expression of the three cytokines in MAC-T cells challenged with LPS (Fig. 2), suggesting anti-inflammatory effects of OPCs on bovine mastitis.

COX-2 and iNOS have also been regarded as inflammatory markers, and PCs have been documented to alleviate LPS-induced neuroinflammation and to downregulate expression of iNOS and COX-2 in mice as well as in hepatocytes around the hepatic portal vein of humans [32, 33]. Actually, COX-2 is an inducible enzyme activated upon cytokines and growth factors, such as IL-1 and IL-6, and induction of COX-2 is a rate-limiting step in prostaglandin biosynthesis; iNOS is a nitric oxide synthase activated by pathologic stimuli, and suppression of iNOS is essential to lower NO levels [10, 34]. Thus, induction of both enzymes is responsible for oxidative stress and occurrence of inflammatory symptoms. In this study, OPCs were found to downregulate production of COX-2 and iNOS in MAC-T cells (Fig. 3), indicating the anti-oxidant effect of OPCs on bovine mastitis.

Transcription factor NF-κB has been well documented as a target to inhibit immunological or inflammatory responses in colitis and macrophages [35, 36]. It exists in the cytoplasm as inactive heterodimeric complexes mainly composed of p50 and p65 subunits binding with inhibitory IκBα [25, 37]. LPS stimulation phosphorylates NF-κB key members IκBα and p65 and allows nuclear translocation of NF-κB p65, thereby activating the transcription of various chemokines and cytokines, such as IL-6, TNF-α, and IL-1β [38, 39]. Accumulating evidence demonstrates natural active ingredients can modulate cellular inflammatory response by inhibiting NF-κB activation [8, 10, 11]. For examples, PCs have been shown to inhibit the production of p-p65 and p-IκB in mouse autoimmune arthritis and macrophages [16, 17]. Our data revealed that OPCs increased abundance of IκB, but lowered phosphorylation of IκB and p65 in LPS-stimulated cells (Figs. 4 and S2), leading to a blockage of p65 translocation to the nucleus (Figs. 6 and S4), suggesting anti-inflammatory effect of OPCs associated with the inhibition of NF-κB activation.

MAPK signals are critical regulators upstream the NF-κB pathway [15]. MAPKs, containing p38, JNK, and ERK, are involved in a cascade regulating NF-κB-mediated transcription of the proinflammatory cytokines IL-6, TNF-α, and IL-1β, and inflammatory mediators, such as NO, iNOS, COX-2, and prostaglandin E2 [15]. Several lines of evidence indicate that PCs directly downregulate the activities of stress-activated MAPK pathway. PCs have demonstrated anti-inflammatory property in mouse brain cells through targeting iNOS and COX-2 as well as IL-6, IL-1β, and TNF-α [15]. Especially, PCs from grape seed, *Pinus massoniana* bark and red rice extract, diminished oxidative stress and enhanced the production and activity of antioxidant enzymes via modulating the activity of NF-κB and the activation of JNK, ERK and p38 MAPK in both in vivo and in vitro studies [15, 26, 40, 41]. The finding that OPCs repressed LPS-induced phosphorylation of ERK, JNK, and p-p38 in MAC-T cells (Fig. 5) indicates the anti-inflammatory and anti-oxidant effects of OPCs via blocking the activities of MAPK pathway.

In conclusion, our data demonstrated that OPC treatment downregulated LPS-induced production of inflammatory cytokines (IL-6, IL-1β, and TNF-α) and oxidative stress indicators (COX-2 and iNOS) in MAC-T cells. The anti-inflammatory activity of OPCs involves the NF-κB and MAPK pathways. Future investigations are needed to test the anti-inflammatory and anti-oxidative efficacy of OPCs in animal models, and to develop novel applications for OPCs in the prevention and treatment of LPS-stimulated mastitis in dairy cows.

## Supplemental Materials



Supplementary data for this paper are available on-line only at http://jmb.or.kr.

## Figures and Tables

**Table 1:** Prime information for real-time PCR.

Gene name	Primer sequence 5′–3′	Size (bp)	Annealing (°C)	Accession No.
TNF-α	F: TCTGGTTCAAACACTCAGGTCC	120	59	NM_173966
	R: AGGGCATTGGCATACGAGTC			
IL-1β	F: AGAGGCAGTTTGGGAGACGA	241	59	NM_174093
	R: GGGACTGGCATGGCAAATGG			
IL-6	F: CACCCCAGGCAGACTACTTC	216	59	NM_173923
	R:AAGCAAATCGCCTGATTGAACC			
iNOS	F: CTGGAGGAAGTGGGCAGAAG	190	59	NM_001076799.1
	R: CTCGGGAGCGGTACTCATTC			
COX-2	F: TAAAGCCAGGGGAGCTACGA	191	59	NC_006853.1
	R: TAAGCCTGGACGGGACGATA			
β-actin	TGGATCAGCAAG	82	59	NM_173979
	R: TAACAGTCCGCCTAGAAGCA			
