# Supplementary material for: Anti-Inflammatory Activity of Oligomeric Proanthocyanidins Via Inhibition of NF-κB and MAPK in LPS-Stimulated MAC-T Cells
Source: J Microbiol Biotechnol. 2020 Aug 28;30(10):1458–66. doi: 10.4014/jmb.2006.06030 (PMC9728330; doi:10.4014/jmb.2006.06030)
Supplement: Supplementary file 1 [file JMB-30-10-1458-supple.pdf]

J. Microbiol. Biotechnol.
https://doi.org/10.4014/jmb.2006.06030

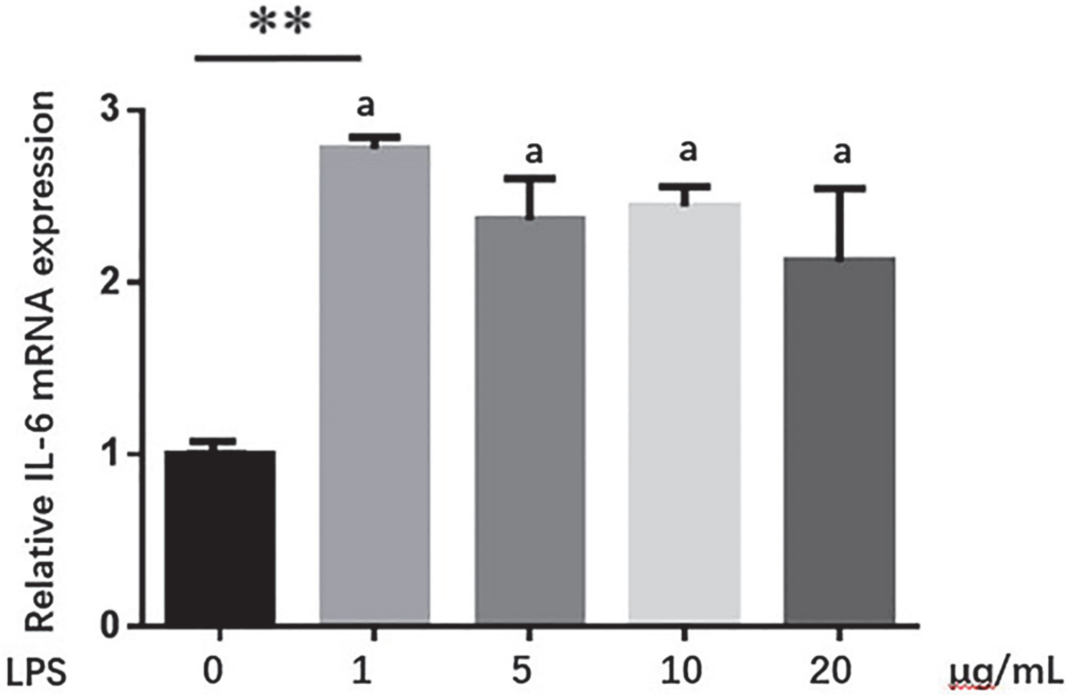

Supplemental Fig.1 Inflammatory effect of LPS in MAC-T cells. Inflammatory effect of LPS in MAC-T cells. Cells were respectively incubated with LPS at 1, 5, 10 and 20 µg/mL for 24 h. Real-time PCR was used to measure IL-6 mRNA levels with β-actin as an internal control. Values represent means ± SEM of four independent experiments. Above bars, ** indicate significance at $P < 0.01$ between the control and the LPS treatments, and the same letter indicates $P > 0.05$ among LPS treatments.

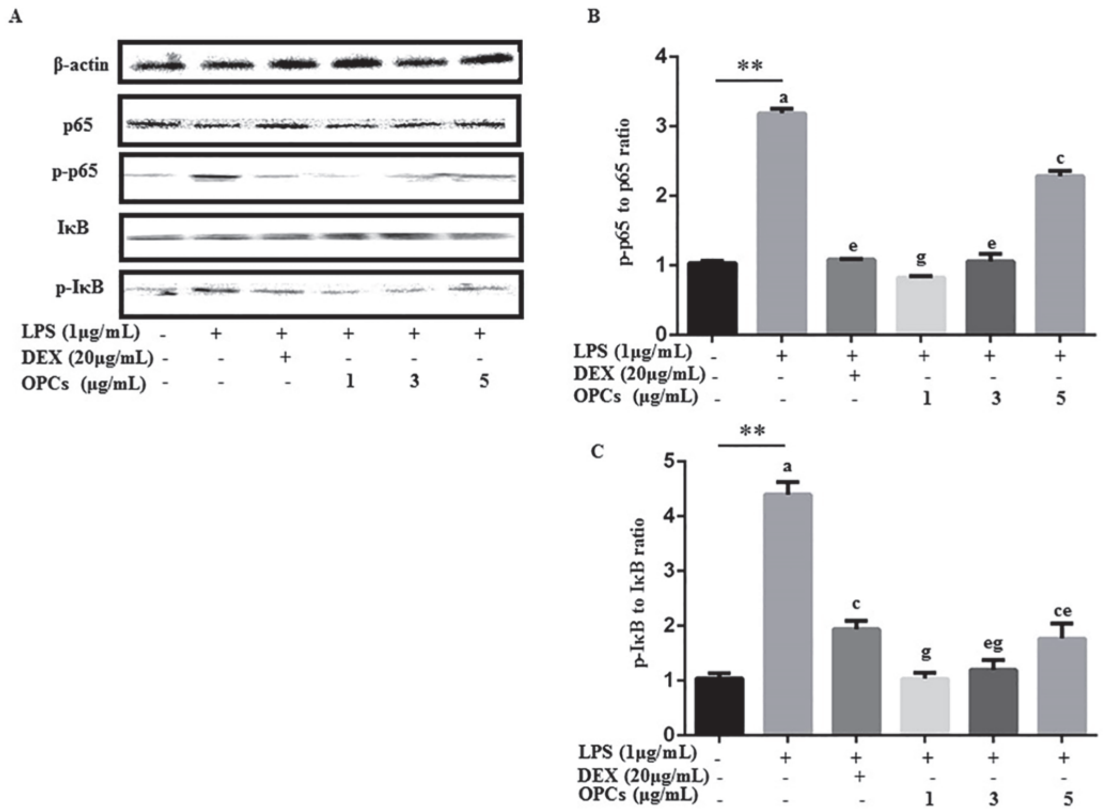

Supplemental Fig. 2 Western blot analysis (A) and p-p65/p65 and p-IκB/IκB ratios (B and C) in MAC-T cells. Cells were incubated with 1 μg/mL LPS for 24 h in combination with DEX (20 μg/mL) or OPCs (1, 3 and 5 μg/mL). Above bars, ** indicate significance at $P < 0.01$ between the control and the LPS treatment without DEX and OPCs. Among LPS in combination with DEX and OPCs treatments, same letters indicate $P > 0.05$, different adjacent letters indicate $P < 0.05$, and different nonadjacent letters indicate $P < 0.01$.

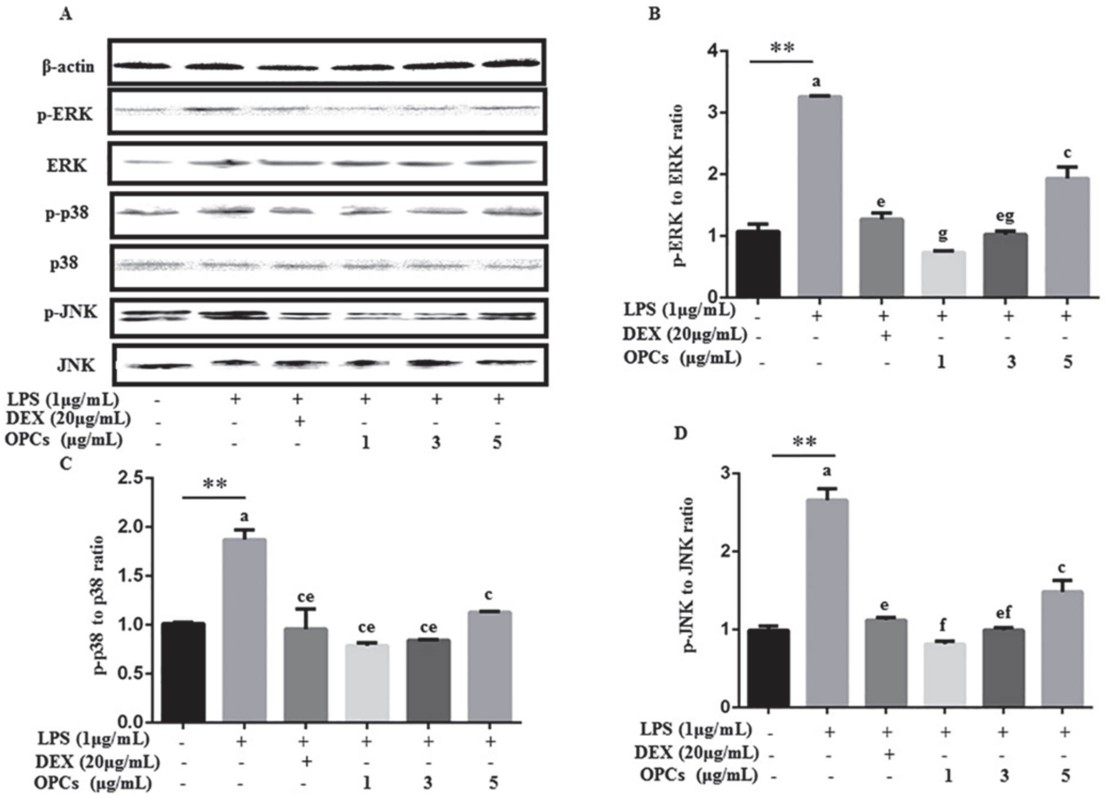

Supplemental Fig. 3 Western blot analysis (A) and ratios of p-ERK/ERK (B), p-p38/p38 (C) and p-JNK/JNK (D) in MAC-T cells. Cells were incubated with 1 μg/mL LPS for 24 h in combination with DEX (20 μg/mL) or OPCs (1, 3 and 5 μg/mL). Above bars, ** indicate significance at $P < 0.01$ between the control and the LPS treatment without DEX and OPCs. Among LPS in combination with DEX and OPCs treatments, same letters indicate $P > 0.05$, different adjacent letters indicate $P < 0.05$, and different nonadjacent letters indicate $P < 0.01$.

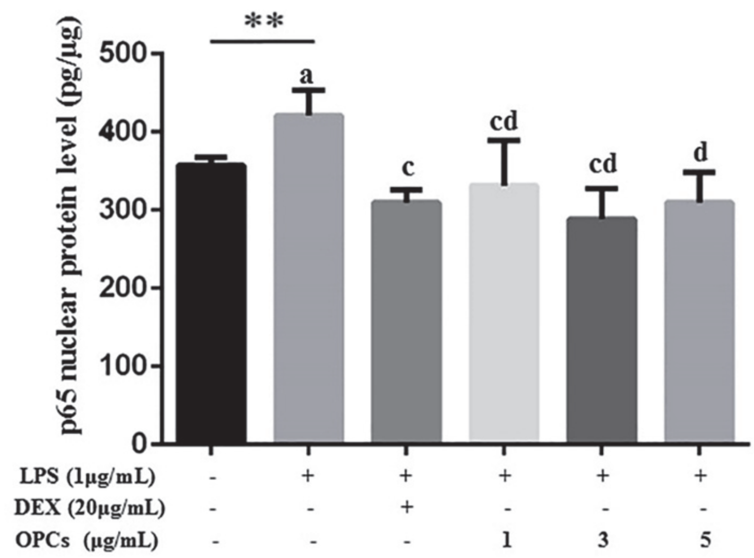

Supplemental Fig.4 Measurement of NF-κB p65 nuclear translocation.

Cells were incubated with 1 μg/mL LPS for 24 h in combination with DEX (20 μg/mL) or OPCs (1, 3 and 5μg/mL). Values represent means ± SEM of four independent. Above bars, ** indicate significance at $P < 0.01$ between the control and the LPS treatment without OPCs. Among LPS in combination with DEX and OPCs treatments, same letters indicated $P > 0.05$, different adjacent letters indicated $P < 0.05$, and different nonadjacent letters indicate $P < 0.01$.
